# Demographic and historical processes influencing *Cochliomyia hominivorax* (Diptera: Calliphoridae) population structure across South America

**DOI:** 10.1186/s13071-024-06622-w

**Published:** 2025-01-20

**Authors:** Kelly da Silva e Souza, Letícia Chiara Baldassio de Paula, Ana Maria Lima de Azeredo-Espin, Tatiana Teixeira Torres

**Affiliations:** 1 Department of Genetics and Evolutionary Biology. Institute of Biosciences, University of São Paulo, Rua Do Matão, 277, São Paulo, SP 05508-090 Brazil; 2 Molecular Biology and Genetic Engineering Center, Campinas State University, Campinas, SP, Brazil

**Keywords:** New World screwworm, Myiasis, Genetic variability, Metapopulation, Isolation by distance, Center–periphery distribution

## Abstract

**Background:**

In this study, we investigated the genetic variability and population structure of the New World screwworm fly *Cochliomyia hominivorax*. We tested the hypothesis that the species exhibits a center–periphery distribution of genetic variability, with higher genetic diversity in central populations (e.g., Brazil) and lower diversity in peripheral populations.

**Methods:**

Using microsatellite markers, we analyzed larvae collected from infested livestock across South America. The larvae were collected directly from various wound sites to ensure a broad representation of genetic diversity.

**Results:**

Contrary to our initial hypothesis, the results revealed consistent genetic variability across the species’ distribution, low population differentiation, and no evidence of isolation-by-distance patterns among subpopulations. The genetic analysis indicated an excess of homozygotes, potentially due to the Wahlund effect, null alleles, or selection pressure.

**Conclusions:**

These findings suggest a complex metapopulation structure for *C. hominivorax*, challenging classical population genetics models. This complexity likely arises from the species’ high dispersal capability and frequent local extinctions followed by recolonization. These results have important implications for the design and implementation of control programs, emphasizing the need for coordinated and large-scale actions rather than isolated initiatives.

**Graphical Abstract:**

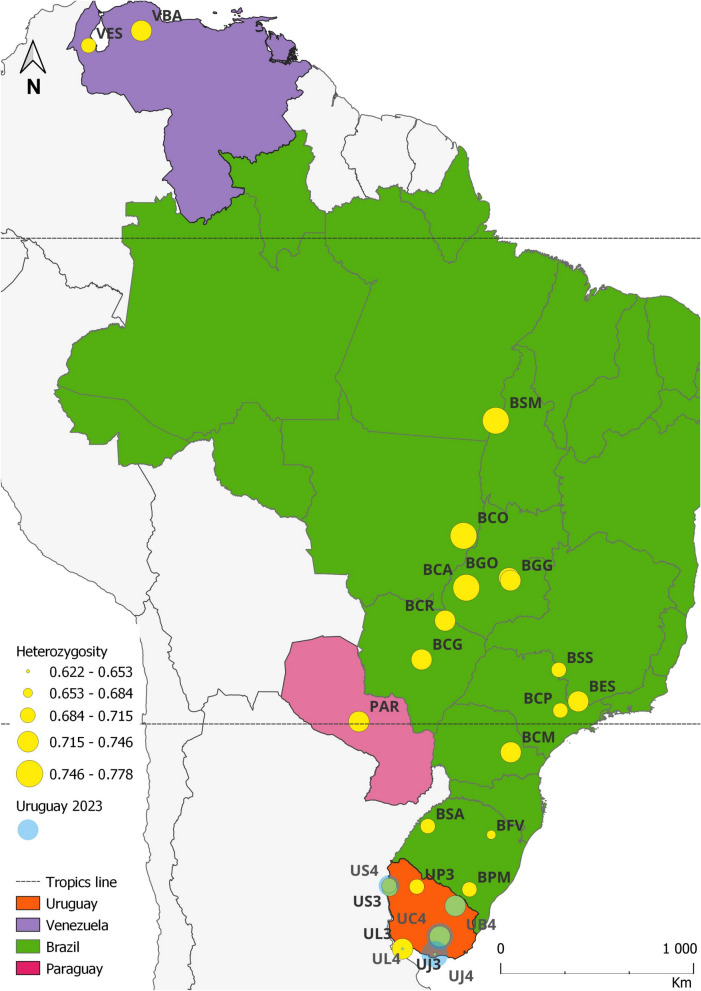

**Supplementary Information:**

The online version contains supplementary material available at 10.1186/s13071-024-06622-w.

## Background

Parasitic diseases significantly influence livestock production, especially in underdeveloped and developing nations [1, 2]. These diseases can lead to a range of detrimental effects on livestock, including increased mortality rates, decreased fertility, weight loss, and reduced milk production, causing a significant direct and indirect impact on production levels [3]. Ecto- and endoparasites are the main culprits, recognized for restricting animal production by compromising the health of cattle, sheep, goats, and other livestock species. Given that over a billion people worldwide rely on livestock, managing and controlling parasitic infections has become a high priority to maintain food security and reduce economic losses [4].

Myiasis, an infection caused by Diptera larvae feeding on living or dead tissues of a live vertebrate host [5], is a significant concern for livestock production. Several fly species have been identified as responsible for some form of myiasis, with the most important species belonging to the families Muscidae, Calliphoridae, Oestridae, and Sarcophagidae [6]. These species have medical, sanitary and economic importance, affecting humans and other animals, while contributing to the spread of pathogens and the increase of agricultural losses [5, 7].

The New World screwworm (NWS) fly *Cochliomyia hominivorax* [8] is an obligate ectoparasite of homeothermic animals [5]. Adult females of this species deposit their eggs in wounds or cavities on the host’s body, with the larvae feeding on living tissue. If not treated, wounds can expand, attracting other females to lay their eggs in the same host [9, 10]. This species is a major pest in most of the Neotropical region, infesting not only domestic animals, primarily cattle, but also wild animals. [11]. While infestation by screwworm larvae can lead to the host’s death in extreme cases, common manifestations include abortion and reduction in milk production, weight, and fertility [12]. Additionally, infestation scars reduce leather quality, affecting its value [13]. Economic losses caused by this species are substantial, encompassing not only reduced productivity and death, but also the costs of manpower and insecticides involved in management, prophylaxis, and treatment. Estimates indicate that costs in South America can reach $3.6 billion annually [14].

Originally, the geographical distribution of the screwworm fly extended from the southern United States to the northern regions of Argentina and Uruguay [7]. Significant economic losses throughout its distribution led to initiatives to control the fly through male sterilization and the implementation of an eradication program, the sterile insect technique (SIT) [15]. The *C. hominivorax* eradication program by SIT began in 1958 in the United States, leading to the country being declared free of cases by 1966. Similar successes were achieved in Mexico and throughout Central America to Panama in 2001, with a permanent barrier maintained at the border between Colombia and Panama to prevent recolonization by flies from South America [16–19]. Despite these efforts, outbreaks such as the one in the Florida Keys in 2016 have occurred, but it was successfully re-eradicated within 6 months [20].

Due to the high cost of SIT, the control of *C. hominivorax* in its current geographical distribution relies on insecticides, mostly organophosphates. However, their extensive use poses serious risks, including toxicity to animals and meat consumers, environmental pollution, and the selection of resistant strains [21–23]*. Cochliomyia hominivorax* is a significant agricultural pest with wide dispersal and adaptability, requiring continuous investment in monitoring, treatment, and control. Research in genetics and ecology is crucial to the development of effective control strategies. A well-established control program can be created by studying the biology, genetic variability, and natural structure of *C. hominivorax* populations. This will contribute to knowledge on the management of NWS, and lead to the development of specific interventions to mitigate its impact on agricultural systems.

### Population genetics of *C. hominivorax*

The first genetic studies on *C. hominivorax* were aimed at characterizing the mutations caused by the exposure of pupae to radiation [24]. Almost 20 years later, genetic studies were resumed using isozymes to characterize the variation caused by pupae irradiation. Bush and Neck [25] identified variations in two loci of enzymes related to energy production for flight, alpha-glycerophosphate dehydrogenase (αGPDH) and phosphoglycerate mutase (PGM), in samples from mass-reared *C. hominivorax*.

McInnis [26], Richardson et al. [27, 28], and Azeredo-Espin [29] initiated studies in North America and Brazil, aimed at the characterization of natural populations with karyotypic, morphological, and sexual compatibility analyses. These studies found great intra- and interpopulation variability in the *C. hominivorax* populations. Dev et al. [30] used cytological analysis of polytene chromosomes from samples of *C. hominivorax* from 10 different localities (nine in Mexico and one in Jamaica) but did not identify reproductively isolated populations. In the late 1980s, molecular markers began to be used to describe the genetic variability and structure of natural populations of *C. hominivorax*.

Initially, mitochondrial DNA (mtDNA) restriction profiles were used to analyze geographical populations of *C. hominivorax* from the United States, Mexico, Costa Rica, Guatemala, and Jamaica [31, 32]. The authors found high genetic variability, significant population structure, and reduced gene flow, mainly between continental samples and samples from the island of Jamaica.

Krafsur and Whitten [33], using three polymorphic isozyme loci, found no population differentiation in 11 geographical samples from Mexico. They concluded that *C. hominivorax* constitutes a single panmictic population. Similar results were found in Costa Rica, indicating the absence of genetic structuring [34]. Comparing previously analyzed samples from Mexico and Costa Rica with samples collected in Rio de Janeiro and Rio Grande do Sul, the authors found no evidence of population structuring [35].

On the other hand, mtDNA analysis of geographical populations of *C. hominivorax* from Brazil revealed a high intra and interpopulation mitochondrial genotypes variability [36, 37]. Studies with restriction fragment length polymorphism (RFLP), random amplified polymorphic DNA (RAPD), and isozymes indicated that the analyzed samples of *C. hominivorax* have high levels of variability and genetic differentiation, indicating population structuring and reduced gene flow [37–39].

Previous studies have shown varied patterns of population structure in *C. hominivorax* across different regions. In Uruguay, research using mtDNA and microsatellite markers found no significant population differentiation, likely due to the absence of geographical barriers and the passive transport of larvae by hosts [40, 41]. In contrast, studies across Central and South America identified moderate structuring, with island populations functioning as independent evolutionary units with significant differentiation and low genetic variability, while mainland populations exhibited high variability and differentiation that, while statistically significant, was relatively low [42]. Additionally, research in the Caribbean using microsatellites revealed moderate structuring among populations, potentially due to limited gene flow or source–sink dynamics [43]. Phylogeographic analyses further highlighted significant genetic divergence across the species’ range, influenced by historical events like Caribbean colonization and Amazonian vicariance, while recent single-nucleotide polymorphism (SNP)-based studies described differentiation levels from moderate to very large throughout its distribution [44, 45].

These previous studies of natural populations of *C. hominivorax* yielded different results, which may appear contradictory. However, a discernible pattern for the distribution of genetic variability in this species can be inferred. In general, these results have indicated a low to moderate genetic structuring in the regions situated at the distribution extremes of this species, contrasting with a high structuring observed in central regions. This pattern suggests a center–periphery distribution of genetic variability. The central populations in Brazil, considered the origin center of the species [39], would harbor older populations and display a pattern of historical differentiation. Populations situated at the distribution peripheries would result from recent colonization events and hence exhibit low levels of population structuring. In this scenario, there would be a population structure following the isolation-by-distance model, with peripheral populations presenting a lower genetic variability than those at the center of the distribution. In light of these observations, our study aimed to investigate the genetic variability and assess the degree of genetic structuring among *C. hominivorax* geographical populations from South America using microsatellite markers, testing the hypothesis of center–periphery distribution of genetic variability.

## Methods

### Obtaining *C. hominivorax* samples from South America

**Fig. 1:**
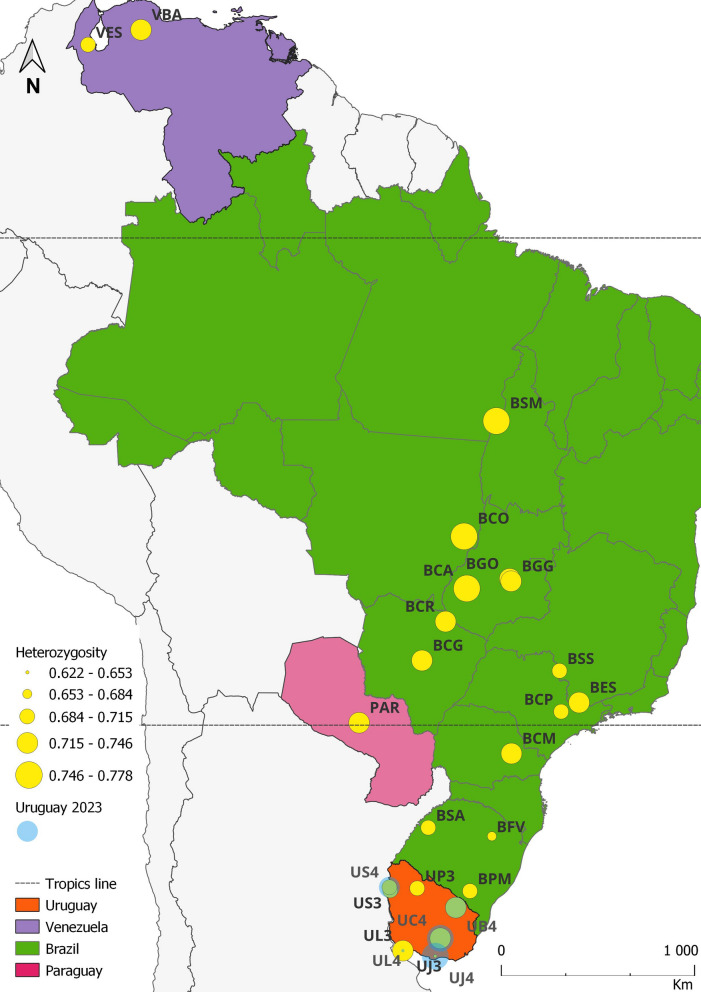
Geographical distribution of *Cochliomyia hominivorax* sample collection sites in South America. Heterozygosity values are indicated by yellow circles, with circle size proportional to heterozygosity levels, ranging from 0.621 in Joaquín Suarez (UJ3) to 0.778 in Caiapônia (BCA). Samples collected in Uruguay on two different dates are marked with a blue fading shading, representing the heterozygosity values for the 2023 sampling event. This shading visually distinguishes temporal variation in heterozygosity at the respective sites

We collected *C. hominivorax* larvae directly from the wounds of infested animals on livestock farms at eight locations in Brazil. To mitigate the sampling bias, which occurs when individuals from a single oviposition are overrepresented due to collecting all larvae from the same wound—potentially resulting in samples representing only one or a few families and causing distortions in the frequency of rare haplotypes or alleles—we implemented criteria to select larvae from different ovipositions, broadening our sampling across wounds from different hosts. Additionally, we used larval stage and mitochondrial haplotype data from previous studies [40, 42] to differentiate individuals on the same wound that originated from different ovipositions. For the remaining locations, local farmers and collaborators collected samples between 2001 and 2006 from wounds of infested animals and shipped them in absolute ethanol (Table 1, Fig. 1).

**Table 1:** Sampled locations with their respective geographical information

Country	ID	Localities	Number of individuals	Latitude	Longitude	Collection date (year)
Uruguay	UD3	Dayman	19	31° 33′ 00 S	57° 57′ 00 W	2003
	US3	San Antonio	21	31° 24′ 00 S	57° 58′ 00 W	2003
	UC3	Cerro Colorado	29	33° 52′ 00 S	55° 33′ 00 W	2003
	UL3	Colonia	32	34° 28′ 00 S	57° 51′ 00 W	2003
	UB3	Banãdos de Medina	24	32° 23′ 00 S	54° 21′ 00 W	2003
	UJ3	Joaquín Suarez	15	34° 44′ 00 S	56° 02′ 00 W	2003
	UP3	Paso Muñoz	15	31° 27′ 00 S	56° 23′ 00 W	2003
	UJ4	Joaquín Suarez	37	34° 44′ 00 S	56° 02′ 00 W	2004
	UL4	Colonia	16	34° 28′ 00 S	57° 51′ 00 W	2004
	US4	San Antonio	20	31° 24′ 00 S	57° 58′ 00 W	2004
	UB4	Banãdos de Medina	16	32° 23′ 00 S	54° 21′ 00 W	2004
	UC4	Cerro Colorado	32	33° 52′ 00 S	55° 33′ 00 W	2004
Brazil	BPM	Pinheiro Machado	24	31° 35′ 00 S	53° 23′ 00 W	2005
	BFV	Fagundes Varela	10	28° 56′ 00 S	51° 41′ 00 W	2005
	BSA	Santo Antonio das Missões	19	28° 31′ 00 S	55° 14′ 00 W	2004
	BCM	Carambeí	37	24° 56′ 00 S	50° 03′ 00 W	2005
	BES	Estiva	47	22° 28′ 00 S	46° 01′ 00 W	2006
	BSS	São Sebastião do Paraíso	17	20° 56′ 00 S	46° 59′ 00 W	2004
	BCG	Campo Grande	12	20° 26′ 00 S	54° 37′ 00 W	2004
	BCR	Costa Rica	12	18° 33′ 00 S	53° 08′ 00 W	2002
	BCA	Caiapônia	42	16° 57′ 00 S	51° 49′ 00 W	2005
	BGO	Goianira	24	16° 29′ 00 S	49° 24′ 00 W	2004
	BGY	Goiânia	20	16° 36′ 00 S	49° 20′ 00 W	2001
	BCO	Cocalinho	15	14° 26′ 00 S	51° 47′ 00 W	2004
	BSM	Santa Maria das Barreiras	46	08° 51′ 00 S	49° 43′ 00 W	2004
	BCP	Campinas	24	22° 54′ 20 S	47° 03′ 38 W	2004
Venezuela	VBA	Barquisimeto	31	17° 18′ 00 S	52° 41′ 00 W	2003
	VMA	Encontrados	30	9° 20′ 51 N	72° 11′ 20 W	2004
Paraguay	PAR	Ybytymí	30	25° 46′ 00 S	56° 41′ 00 W	2004

### Analysis of microsatellite data

#### Detection of microsatellite loci polymorphism

To analyze the geographical populations of South America, we used 12 loci selected from previous work [46, 47]. We performed polymerase chain reaction (PCR) amplification and visualization of the microsatellite loci as detailed in Additional file 1: Table S1, following the methods described by Torres et al. ([46]; Table S1).

We determined the number and frequency of alleles, as well as the observed heterozygosity (*H*_O_) and unbiased expected heterozygosity (*H*_E_) under Hardy–Weinberg equilibrium for each locus and location. We used the *basic.stats* function from the R package *hierfstat* version 0.5.10 [47] to calculate the average *H*_O_, *H*_E_, and inbreeding coefficient (*F*_IS_). To assess deviations from Hardy–Weinberg equilibrium expectations, we applied exact tests for each locus and population in GENEPOP 4.7 [48, 49].

We used allele frequency data and applied statistical tests with BOTTLENECK 1.2.02 to detect signatures of heterozygosity excess (*H*_Exc_) at all microsatellite loci, indicating a potential recent bottleneck event [50]. We employed the sign test to evaluate the number of loci exhibiting heterozygosity excess compared to the expected number by chance under various mutational models, including the infinite alleles model (IAM), the stepwise mutation model (SMM), and the two-phase model (TPM). For the TPM, we used proportions favoring IAM, specifically 30% for SMM and 70% for IAM.

We measured interpopulation differentiation in South American samples using *F*_ST_ estimates. We used three sets of subpopulations to calculate interpopulation differentiation, including *F*_ST_ estimation, the inbreeding coefficient *F*_IS_, and the estimated number of migrants *Nm*. The first set includes all samples from Table 1, excluding only samples obtained from Uruguay in 2004. The second set excludes only samples obtained from Uruguay in 2003. The third group includes all samples from Table 1, comprising both samples from Uruguay in 2004 and 2003 without grouping them. We calculated the genetic differentiation index, *F*_ST_, between localities using absolute allele frequencies, following the methodology of Weir and Cockerham [51], and implemented it with the *hierfstat* library in R [47]. To assess the significance of pairwise *F*_ST_ estimates, we performed genotype permutation among populations, as described by Goudet et al. [52]. The critical significance level applied in all statistical tests was 0.05. We corrected the critical significance levels in all simultaneous statistical tests using the sequential Bonferroni test [53] to enable the overall significance to be examined. We evaluated the isolation-by-distance model, which assesses population genetic structure, using linear regression. This involved correlating pairwise *F*_ST_/(1 − *F*_ST_) with the natural logarithm of the geographical distance between population pairs [54]. Additionally, we compared the genetic variability between sample groups using Wilcoxon signed-rank tests. We performed these analyses on the R [55] computational platform.

## Results

### Genetic variability

The number of alleles of *H*_O_ and *H*_E_ were calculated per locus and population (Additional file 2: Table S2; Additional file 3: Figure S1). The number of alleles detected per locus ranged from 2 to 18, with an average of 6.9 alleles per locus and per population. The *H*_E_ ranged from 0.194 (Costa Rica, Brazil, locus CH09) to 0.936 (Costa Rica, Brazil, locus CH21), with an overall average of 0.722. With the exclusion of samples from Uruguay, the average did not change, and it was 0.727. Significant deviations from the Hardy–Weinberg equilibrium were found in 187 out of 348 tests, though after sequential Bonferroni correction, this number was reduced to 124 significant tests. Such deviations were consistent across all samples analyzed and were present in at least one locus per location. Linkage disequilibrium analysis revealed that 376 out of 1782 comparisons between loci pairs showed linkage disequilibrium (*P* < 0.05). Additionally, the analysis revealed non-random associations between loci pairs in 56 comparisons after sequential Bonferroni correction. These imbalances varied across subpopulations.

Analysis of all loci revealed the lowest variability in *H*_E_ in Colonia del Sacramento, Uruguay (2004), with *H*_E_ equal to 0.633 (Fig. 1), significantly different from the overall mean (Wilcoxon signed-rank test, *P* = 0.001). Similarly, Joaquín Suarez, Uruguay (2003), exhibited lower mean variability (*H*_E_ = 0.622; Wilcoxon signed-rank test, *P* = 0.0243). The highest observed variability was recorded in Joaquín Suarez, Uruguay (2004) (0.774; *P* = 0.02), Santa Maria das Barreiras, Brazil (0.775; *P* = 0.0018), and Caiapônia, Brazil (0.778; *P* = 0.0001). Heterozygosity was also significantly different from the overall mean in the populations of San Antonio, Uruguay (2003 and 2004), Cerro Colorado, Uruguay (2004), and Campinas, Brazil (Additional file 2: Table S2). Other observed differences from the mean were not statistically significant.

To test the decrease in genetic variability at the extreme south of *C. hominivorax* distribution, we compared the *H*_E_ of the southern populations, below the Tropic of Capricorn (subpopulations of Uruguay and four subpopulations of southern Brazil) with populations of the central region (the remaining Brazil and Venezuela subpopulations). *Cochliomyia hominivorax* variability in the south (*H*_E_ = 0.7206) was not significantly different from the variability of central populations (*H*_E_ = 0.7372), with *P* = 0.251. Additionally, no significant difference was observed when comparing allelic diversity (“allelic richness,” DA) between southern and central groups (*DA*_south_ = 4.71; *DA*_central_ = 4.94; *P* = 0.10987) or when considering only samples from Uruguay as a southern group (*H*_E Uruguay_ = 0.7206, *H*_E central_ = 0.7372 and *P* = 0.5658; *DA*_Uruguay_ = 4.84, *DA*_central_ = 4.94 and *P* = 0.16967).

A bottleneck was not detected in any of the subpopulations across all three mutation models (Table 2). However, in all of them except San Antonio, Uruguay (2003), Santo Antonio das Missões, and São Sebastião do Paraíso, Brazil, an excess of heterozygotes was detected in relation to the number of heterozygotes expected at equilibrium in the IAM model. Under the SMM model, nine populations putatively experienced a reduction in population size (San Antonio and Banãdos de Medina 2003, Pinheiro Machado, Santo Antonio das Missões, Carambeí, Estiva, São Sebastião do Paraíso, Caiapônia, and Goiânia). Under TPM, there was a significant population reduction in San Antonio (2004) and Cerro Colorado (2004).

**Table 2:** Tests to detect the populational reduction on the BOTTLENECK program

ID	Mutation model	*P*-value		Heterozygote interpretation
		*H*_Def_	*H*_Exc_	
UD3	IAM	0.99329	0.01709*	Excess
	TPM	0.63330	0.39551	Equilibrium
	SMM	0.15063	0.86694	Equilibrium
US3	IAM	0.86694	0.15063	Equilibrium
	TPM	0.23486	0.78809	Equilibrium
	SMM	0.02124*	0.98291	Deficiency
UC3	IAM	0.99597	0.00525*	Excess
	TPM	0.78809	0.23486	Equilibrium
	SMM	0.08813	0.92432	Equilibrium
UL3	IAM	0.99915	0.00122*	Excess
	TPM	0.95386	0.05493	Equilibrium
	SMM	0.42505	0.60449	Equilibrium
UB3	IAM	0.97388	0.03198*	Excess
	TPM	0.57495	0.45483	Equilibrium
	SMM	0.02612*	0.97876	Deficiency
UJ3	IAM	0.99329	0.01709*	Excess
	TPM	0.92432	0.08813	Equilibrium
	SMM	0.25928	0.76514	Equilibrium
UP3	IAM	0.99475	0.00671*	Excess
	TPM	0.78809	0.23486	Equilibrium
	SMM	0.33862	0.68896	Equilibrium
UJ4	IAM	1.00000	0.00012*	Excess
	TPM	0.95386	0.05493	Equilibrium
	SMM	0.08813	0.92432	Equilibrium
UL4	IAM	0.99475	0.00671*	Excess
	TPM	0.71533	0.31104	Equilibrium
	SMM	0.15063	0.86694	Equilibrium
US4	IAM	0.99988	0.00024*	Excess
	TPM	0.96802	0.03857*	Excess
	SMM	0.25928	0.76514	Equilibrium
UB4	IAM	0.99329	0.01709*	Excess
	TPM	0.76514	0.25928	Equilibrium
	SMM	0.16968	0.84937	Equilibrium
UC4	IAM	0.99939	0.00085*	Excess
	TPM	0.97388	0.03198*	Excess
	SMM	0.11670	0.89819	Equilibrium
BPM	IAM	0.96802	0.03857*	Excess
	TPM	0.42505	0.60449	Equilibrium
	SMM	0.03857*	0.96802	Deficiency
BFV	IAM	0.98291	0.02124*	Excess
	TPM	0.83032	0.19019	Equilibrium
	SMM	0.48486	0.54517	Equilibrium
BSA	IAM	0.80981	0.21191	Equilibrium
	TPM	0.16968	0.84937	Equilibrium
	SMM	0.00171*	0.99878	Deficiency
BCM	IAM	0.99695	0.00403*	Excess
	TPM	0.71533	0.31104	Equilibrium
	SMM	0.03857*	0.96802	Deficiency
BES	IAM	0.99878	0.00171*	Excess
	TPM	0.54517	0.48486	Equilibrium
	SMM	0.00305*	0.99768	Deficiency
BSS	IAM	0.91187	0.10181	Equilibrium
	TPM	0.36670	0.66138	Equilibrium
	SMM	0.04614*	0.96143	Deficiency
BCG	IAM	0.99329	0.01709*	Excess
	TPM	0.84937	0.16968	Equilibrium
	SMM	0.15063	0.86694	Equilibrium
BCR	IAM	0.99829	0.00232*	Excess
	TPM	0.96802	0.03857*	Excess
	SMM	0.68896	0.33862	Equilibrium
BCA	IAM	0.99878	0.00171*	Excess
	TPM	0.78809	0.23486	Equilibrium
	SMM	0.00305*	0.99768	Deficiency
BGO	IAM	0.99475	0.00671*	Excess
	TPM	0.95386	0.05493	Equilibrium
	SMM	0.21191	0.80981	Equilibrium
BGY	IAM	0.99988	0.00024*	Excess
	TPM	0.63330	0.39551	Equilibrium
	SMM	0.01709*	0.99329	Deficiency
BCO	IAM	0.99963	0.00061*	Excess
	TPM	0.98291	0.02124*	Excess
	SMM	0.42505	0.60449	Equilibrium
BSM	IAM	0.99988	0.00024*	Excess
	TPM	0.91187	0.10181	Equilibrium
	SMM	0.08813	0.92432	Equilibrium
BCP	IAM	0.99963	0.00061*	Excess
	TPM	0.99329	0.01709*	Excess
	SMM	0.76514	0.25928	Equilibrium
VBA	IAM	0.96802	0.03857*	Excess
	TPM	0.78809	0.23486	Equilibrium
	SMM	0.05493	0.95386	Equilibrium
VMA	IAM	0.99768	0.00305*	Excess
	TPM	0.80981	0.21191	Equilibrium
	SMM	0.33862	0.68896	Equilibrium
PRA	IAM	0.99988	0.00024*	Excess
	TPM	0.99939	0.00085*	Excess
	SMM	0.93530	0.07568	Equilibrium

Mutation models tested include IAM (infinite alleles model), TPM (two-phase model), and SMM (stepwise mutation model). *H*_Def_ represents heterozygote deficiency, while *H*_Exc_ indicates heterozygote excess. The table highlights whether the population shows “excess,” “equilibrium,” or “deficiency,” providing insights into past demographic events

* Indicates significance at *P* < 0.05

### Genetic differentiation

The genetic differentiation index (*F*_ST_) between subpopulation groups was statistically significant but low, suggesting a mild population structure in the analyzed samples. The inbreeding coefficient (*F*_IS_) showed moderate values, as did the estimated number of migrants (Nm) among groups. These values are surprisingly low for a species with a high dispersal rate, especially considering the vast territorial range covered, with samples collected up to 5000 km apart (Table 3).

**Table 3:** Global estimates in the sampled groups

	Estimated *F*_ST_ (*θ*)	*F*_IS_	*Nm*^a^
Group 1 (24 subpopulations)	0.037*	0.210	*3.31*
Group 2 (22 subpopulations)	0.046*	0.231	*3.35*
Group 3 (29 subpopulations)	0.044*	0.220	*2.74*

Genetic differentiation (*F*_ST_) (θ), inbreeding coefficients (*F*_IS_), and number of migrants between subpopulations (*Nm*)

* *P* < 0.001

^a^Estimated number of migrants after correction for population size

**Fig. 2:**
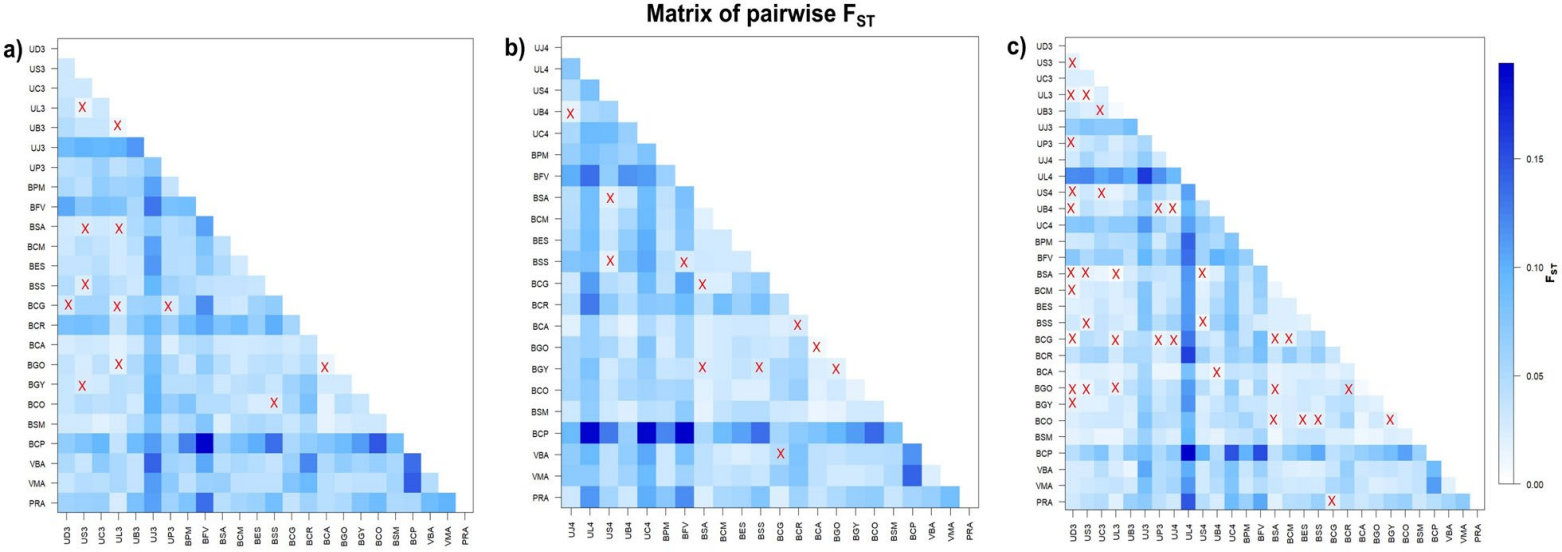
Heatmap matrix representation of pairwise *F*_ST_ estimates between subpopulations for three different population groups. Each matrix shows *F*_ST_ estimates, with color intensity ranging from white (low *F*_ST_) to dark blue (high *F*_ST_). **a** Group 1, excluding samples from Uruguay in 2004. **b** Group 2, excluding samples from Uruguay in 2003. **c** Group 3, including all samples from Uruguay in 2003 and 2004, without grouping them. Non-significant pairwise *F*_ST_ estimates are marked with an “x”

The* F*_ST_ pairwise estimates were generally low and different from zero for 264 out of 276 pairs within group 1 (Fig. 2a), and for 220 out of 231 pairs within group 2 (Fig. 2b). For group 3, the estimates were also different from 0 for 371 out of 406 pairs, ranging from 0.00686 (between Colonia, Uruguay 2003 and Banãdos de Medina, Uruguay 2003) to 0.13815 (between Joaquín Suarez, Uruguay 2003 and Colonia, Uruguay 2004). Nine subpopulations that presented all significant estimates in group 3 (Joaquín Suarez, Uruguay 2004; Colonia, Uruguay 2004; San Antonio Uruguay 2003, 2004; Banãdos de Medina 2004; Cerro Colorado 2003, 2004; Campinas, Brazil; Barquisimeto and Encontrados, Venezuela) differed from all other populations. Nevertheless, estimates were low, ranging from 0.0251 (with São Sebastião do Paraíso) to 0.1193 (with Colonia del Sacramento (2004), Uruguay) (Fig. 2c).

**Fig. 3:**
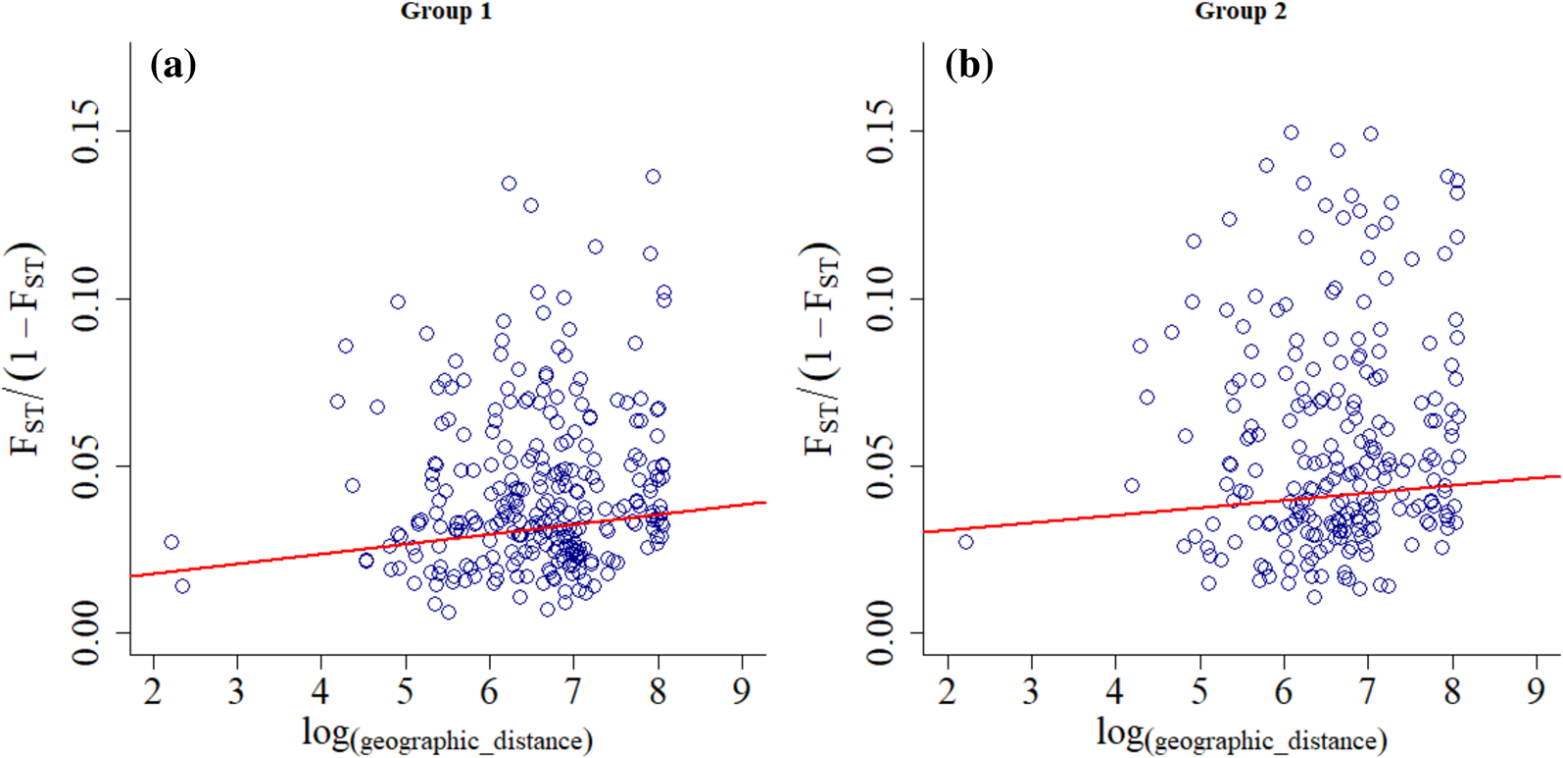
Test for isolation by distance (IBD) in two population groups. Linear regression of *F*_ST_/(1 − * F*_ST_) between population pairs against the natural logarithm of geographical distances between population pairs was performed for **a** group 1 subpopulations and **b** group 2 subpopulations. Mantel tests revealed no significant correlation between genetic and geographical distances for either group

Mantel tests showed no significant correlation between the genetic and geographical distances from samples in group 1 (*P* = 0.1954, Fig. 3a) or in group 2 (*P* = 0.137, Fig. 3b). This pattern did not change even after excluding locations separated by distances less than 100 km (group 1, *P* = 0.116; group 2, *P* = 0.158) or 200 km (group 1, *P* = 0.134; group 2, *P* = 0.152). Tests using other minimum and maximum distances also failed to indicate any genetic and geographical distance association (data not shown).

The low differentiation found between subpopulations was not expected at such large distances (for example, *F*_ST_ = 0.0534 estimation between samples presenting the greatest geographical distance, 5180 km).

We observed temporal sub-structuring of *C. hominivorax* subpopulations in the southern region of Uruguay, specifically in Joaquín Suarez and Colonia. These populations exhibit significant differences in heterozygosity within the same locations across different years (*H*_E_ for UJ3 = 0.66 and UJ4 = 0.82; UL3 = 0.77 and UL4 = 0.63), with greater differentiation (*F*_ST_ pairwise) compared to other locations (*F*_ST_ pairwise UJ3 and UJ4 = 0.074; UL3 and UL4 = 0.095).

## Discussion

According to our initial hypothesis, based on previous studies on *C. hominivorax* populations, we anticipated a high degree of genetic differentiation in subpopulations with a central distribution, structured according to an isolation-by-distance model. Additionally, peripheral populations were expected to exhibit reduced genetic variability compared to central populations. However, we found that (1) genetic variability remained consistent across the species’ distribution extremes, (2) population differentiation was low and (3) subpopulations did not follow an isolation-by-distance model, and (4) temporal fluctuations were evident in peripheral populations.

Our results contrast with those obtained by Infante-Vargas and Azeredo-Espin [38] and Infante-Malachias [37]. Using different molecular markers, such as mtDNA, RFLP markers, RAPD, and isozymes, they indicated high levels of genetic differentiation with reduced gene flow among Brazilian *C. hominivorax* populations. In contrast, Lyra et al. [42] found lower, but still significant, differentiation among mainland populations using mtDNA PCR–RFLP. The discrepancies between these studies might be due to differences in the molecular markers used and the sampling locations.

In addition to differences in molecular markers, the sampling methodology is key distinction between our study and previous ones. Lyra et al. [42] and we both collected larvae directly from infested animals to overcome the challenges of using artificial attractants for collecting adults of *C. hominivorax*. However, this approach could introduce bias if larvae from a single oviposition are overrepresented. For example, larvae from a single wound may come from only one or a few females, which could lead to sampling biases. In previous studies, between 16 and 27 individuals were sampled from one to nine wounds per subpopulation, which may have resulted in an overrepresentation of rare haplotypes or alleles. To address this, we implemented stricter criteria by broadening our sampling across wounds from different hosts and incorporating mitochondrial haplotypes to differentiate larvae from different ovipositions on the same wound.

In the current analysis using all loci together, all samples exhibited an excess of homozygotes compared to the expected equilibrium, potentially attributable to different factors, including the Wahlund effect, null alleles, and selection pressure [56]. First, these deviations can stem from the Wahlund effect [57], which arises when a population is not homogeneous but consists of subpopulations with varying allele frequencies [58]. When samples from these subpopulations are mixed, it can lead to an excess of homozygotes compared to what would be expected if all individuals were mating randomly [59, 60]. This was confirmed by the positive correlation observed between *F*_IS_ and *F*_ST_.

The excess of homozygotes could also be due to the presence of null alleles, which might lead to an underestimation of heterozygosity [61]. Although null alleles may be present at some loci, this does not seem to be the only one responsible for the excess of homozygotes, since the deviations were generally observed in at least one locus in all subpopulations. Furthermore, there was no change in these results with the exclusion of the locus with the highest null alleles estimate (CH10, data not shown). Once again, the occurrence of demographic changes appears as a possible explanation for the results.

Lastly, the excess of homozygotes may be due to selection acting on one or more loci; if one or more loci are under selection pressure, it can lead to an excess of homozygotes if certain alleles are being favored over others [62]. The control of *C. hominivorax* with insecticides has caused rapid selection for resistance in some populations [21–23]. This resistance is multilocus, potentially affecting the entire genome and influencing a wide range of genetic traits and interactions [23]. Baqir and Ab Majid [63] highlight the selection pressure in the population genetic structure of tropical bed bugs in Iraq. Their study reveals bottleneck events with an excess of homozygosity, suggesting a decrease in the effective size of the tropical bed bug population due to control activities and the speed at which the population recovered, emphasizing the role of selection in these populations.

Further investigation is needed to elucidate the underlying cause of the observed excess of homozygotes. Understanding this phenomenon is crucial for accurately interpreting population genetic dynamics. Therefore, our interpretation of *C. hominivorax* population structure and dynamics focuses on two main areas: (A) historical processes and (B) contemporary demographic processes.

### A. Historical processes

Estimates of gene flow between species populations are generally based on differentiation indices such as *F*_ST_ estimates [64, 65]. This approach relies on several assumptions, the most crucial of which is the balance between mutation, migration, and genetic drift in many natural populations. This balance is often disrupted, and such violations are especially critical in pest species, as these species generally have high effective population sizes and a relatively recent demographic history [66–68].

The low genetic structure observed in *C. hominivorax* populations, indicated by low *F*_ST_ values and the lack of isolation by distance, can likely be explained by a recent population expansion. Similar genetic patterns have been documented in populations of other insect species that rely on domesticated hosts, including *Anopheles gambiae* [69]*, Anopheles arabiensis* [70], *Ceratitis capitata* [71], *Ceratitis rosa* and *Ceratitis flaviventris* [72]. These patterns were also observed in *Drosophila melanogaster* [73], and in populations of dung beetles of the species *Aphodius fossor* that uses bovine manure to feed the larvae, demonstrating a high dependence on livestock [74].

A parallel can be drawn between these studies and our analysis of *C. hominivorax* populations. In our study, a deviation from the Hardy–Weinberg equilibrium was observed in nearly every test performed, alongside a high number of significant linkage disequilibrium results. These findings also support the hypothesis of a recent population expansion in *C. hominivorax*.

The genus *Cochliomyia* consists of four species, with *C. hominivorax* as the only obligate ectoparasite among them [75]. Records of *Cochliomyia* species across the Caribbean and parts of Central America indicate a broad geographical range; however, previous studies suggest that South America is likely the center of diversity and origin for *C. hominivorax* [37]. A recent population expansion could be associated with the introduction of livestock, such as cattle, in South America. The first record of the introduction of cattle in South America dates back to 1524 in the present-day Colombia [76], and by 1614, livestock farming had become a standard practice, impacting the population dynamics of *C. hominivorax* in rural areas. This introduction created an increase in host availability, facilitating the expansion in both population size and distribution area for *C. hominivorax*. This expansion likely enhanced connectivity between subpopulations, reducing genetic differentiation by homogenizing genetic diversity through continuous gene flow. Such expansion would have disrupted the equilibrium between migration and genetic drift, reducing genetic structure in *C. hominivorax*, a phenomenon also observed in other species undergoing recent demographic expansions [66–68]. While additional studies are needed to confirm these findings, the historical context supports the hypothesis that population growth, driven by the expansion of host availability, contributed to the lack of genetic structure observed in *C. hominivorax*.

Regardless of which scenario best describes the evolutionary history of this species, the human influence on the population structure of this livestock pest and its potential for dispersion and adaptation is evident. Therefore, *C. hominivorax* should be considered a high-risk species when introduced into a new environment.

### B. Contemporary demographic processes

Analyses of South American samples yielded results similar to those observed in the Uruguayan samples [41]. The migration of adult flies between different subpopulations is not enough to explain the low population differentiation, since this would result in a positive correlation between geographical and genetic distances.

Passive migration of larvae through infested animals could explain, in part, the observations at a local scale. The transport of animals can occur between different farms of the same owner or in the early stages of infestation (such as eggs or first-instar larvae) with animal trade. However, passive migration would not cause the observed patterns at the analyzed scale, for several reasons: (1) the cost of transporting animals over long distances is very high, restricting this practice; (2) infestation in more advanced stages is easy to detect, which makes it challenging to trade infested animals; and (3) inspection is more restricted at international borders.

Fountain et al. [67] used 21 microsatellite markers to investigate human-facilitated metapopulation dynamics in *Cimex lectularius*, a bed bug emerging pest. They emphasize the impact of colonizer numbers and demographic history on genetic diversity distribution. Pest control leads to local extinctions, while human-facilitated dispersal fosters colonization, shaping metapopulation dynamics. Founder events reduce diversity and increase genetic drift, causing rapid population divergence. Despite low diversity within infestations, genetic differentiation among infestations (*F*_ST_ = 0.59) highlights a high population structure.

While the acknowledgment of the influence of time on the genetics of animal populations has expanded through the inclusion of time as a variable in research and analysis [77–79], our endeavor represents a pioneering step in integrating the concept of “isolation by time” into genetic analyses of *C. hominivorax* populations. The results obtained here with the temporal analysis, comparing samples in Uruguay at the same location in two consecutive years, indicate that the local population dynamics of the screwworm fly include population fluctuations dependent on the occurrence of migration, extinction, and recolonization. Temporal genetic differentiation can occur when local extinction occurs, due to significant fluctuations in population size and subsequent colonization of the empty habitat by individuals from populations from more distant locations. Thus, the data obtained fit into a metapopulation model. At the population level, barriers to dispersal and regional selection play a crucial role in shaping the metapopulation configuration, thereby influencing evolutionary dynamics [80].

A metapopulation can be defined as a set of subpopulations of a given species, with each subpopulation occupying a different fragment of a subdivided habitat. Most species are naturally distributed as metapopulations [81, 82]: many insects live in trees or other plants, which are a fragment of habitat; many fish and marine invertebrates live on coral reefs; most parasites live on hosts that are effectively fragments of habitat. In the specific case of *C. hominivorax*, there is a fragmentation of habitats in several aspects: each farm animal where the larvae develop, as well as each livestock region, can be considered a fragment, which usually are not continuous, with areas of agricultural production that could act as a form of fragmentation of habitat. Finally, the distribution of forests where there are sites of aggregation of adult flies [83] is currently fragmented due to human action. Thus, according to species distribution in fragmented habitats and the results demonstrated here, each subpopulation of *C. hominivorax* should be seen as an integral part of a metapopulation. For other species-sharing features with *C. hominivorax*, such as wide distribution and pest status, population structures have already been described using the metapopulation model. Evidence indicates that populations of the blowfly *Phormia regina* exhibit pronounced temporal structure that seems to correspond with seasonal shifts, suggesting that *P. regina* likely operates within a metapopulation framework. Owings et al. [79] stress the significance of spatiotemporal sampling in uncovering the population genetic structure in blowflies, alongside the impact of abiotic variables on these patterns. Their study delved into the temporal population genetic structure of nine populations of *P. regina* over 3 years, analyzing them at six polymorphic microsatellite loci. The findings suggest a robust temporal structure mirroring seasonal variations, indicative of a metapopulation dynamic. Molecular variance analysis of these populations corroborated significant temporal genetic differentiation, while further analyses revealed correlations between abiotic factors like temperature, humidity, precipitation, and wind speed with the observed genetic subdivisions.

This population dynamics model has been observed in several insect species, where migration and recolonization play key roles in maintaining populations. For instance, *Ceratitis capitata* in Israel exhibits a similar recovery pattern, where it recolonizes from more favorable areas during the summer after winter die-off [84]. Similarly, studies on *Plutella xylostella* in southern China and Southeast Asia [85], and *Schistocerca gregaria* in the Old World [86], reveal large-scale migration and metapopulation dynamics that facilitate population persistence. These findings align with observations on *Aedes aegypti* populations in Manaus, Brazil, where seasonal shifts in gene flow and population size also emphasize the importance of recolonization and migration in maintaining pest populations [87]. These studies highlight the critical role of temporal dynamics and migration in pest management and population sustainability.

### Implications for control measures

Generally, population genetics models ignore the complexity of biological systems. Evolutionary estimates parameters, such as gene flow, are rarely valid because the parameters that define these models are spatially and temporally variable and are very sensitive to evolutionary forces. Conventional models assume that population structure and demographic parameters such as population size and dispersion rates are uniform and constant in time and space. Demographic and genetic balance assumptions are unrealistic and violated, as verified in *C. hominivorax* populations in South America. The *C. hominivorax* population structure does not follow classical population genetics models.

Our data reveal a new scenario much more complex than what had been imagined for *C. hominivorax*. Thus, the implications for the establishment of control programs are not intuitive. Each new hypothesis carries distinct implications for control measures, such as the SIT, which may require adjustments in the selection and release of sterile males; biological control strategies, which might require regional adaptations due to varying pesticide sensitivity; and monitoring programs, which would benefit from incorporating the genetic and behavioral diversity among subpopulations.

The recolonization after population reduction in the adverse season is likely accompanied by a rapid *C. hominivorax* population growth rate that may have ensured the maintenance of genetic variability. This is consistent with the species recovery capacity that suffers a drastic population reduction in the dry season and recovers quickly in the rainy season. This seasonality in the screwworm fly infestation cases is observed throughout its distribution. Another common factor among the analyzed locations is the indiscriminate use of insecticides, which would also cause this fly’s population reduction.

No previous studies have investigated the dynamics and genetic structure of *C. hominivorax* populations prior to control programs using SIT, making it difficult to predict outcomes of similar programs in South America or the Caribbean islands. If the scenario proposed in this study is confirmed by future research, it is likely that the most effective strategy for controlling *C. hominivorax* populations would require coordinated action across multiple countries, rather than independent efforts by individual nations or farmers. In Brazil, for instance, control is often carried out by livestock owners applying pesticides on an as-needed basis, without a national coordinated program. A synchronized, broad-scale approach may offer a more effective means of controlling this species. Additionally, the success of any control program will depend on an integrated system that combines various control methods, with strategic timing during periods of reduced population density, typically linked to seasonal climate variations. Targeting cooler periods, when populations are reduced and concentrated in warmer refuges, could prevent these areas from acting as sources for recolonization, which would otherwise compromise the effectiveness of the eradication initiative.

Here we provide essential data on the genetic population structure for this species that, together with information obtained from ecological studies, can be used to assess the necessary size of the management unit and the program implementation area, providing fundamental information for decision-making regarding the eradication of this important pest of livestock throughout its current geographical range.

## Conclusions

The effective size of the *C. hominivorax* metapopulation in South America is large and dynamic, ensuring the preservation of genetic diversity and reducing the risk of local extinction due to processes such as inbreeding. The metapopulation has a high probability of survival due to gene flow and recolonization, with migration between subpopulations maintaining overall stability. This stability persists even if some subpopulations undergo local extinctions during cold and dry periods or due to local pesticide use. Although the temporal analysis included only a few populations over a short time period, this novel finding, supported by the metapopulation structure of the species, indicates that control strategies should be multifaceted. They should not only focus on adult population management, but also address seasonal refuges where the species may survive in a dormant state during colder months. In warmer seasons, strategies should include reducing breeding sites and disrupting the development stages of larvae and pupae. Identifying and managing these refuges during the cold season is crucial to preventing a resurgence when conditions improve. Consequently, a coordinated approach involving environmental management, targeted chemical treatments, and continuous monitoring is essential for effective year-round control. Effective and sustainable pest control must consider the connectivity between subpopulations to optimize management efforts. Based on our results, we recommend synchronized control strategies across countries, focusing not only on seasonal efforts during the species’ reproductive period, but also on detecting refuges to prevent repopulation in already controlled areas.

## Supplementary Information

Additional file 1: Table S1. Microsatellite loci polymorphism.

Additional file 2: Table S2. Genetic diversity of *C. hominivorax* samples collected in different locations in South America.

Additional file 3: Figure S1. Number of alleles.

## Data Availability

No datasets were generated or analyzed during the current study.
